# Robotic- and orthosensor-assisted versus manual (ROAM) total knee replacement: a study protocol for a randomised controlled trial

**DOI:** 10.1186/s13063-021-05936-9

**Published:** 2022-01-22

**Authors:** Nick D. Clement, Michelle Bardgett, Steven Galloway, Y. Jenny Baron, Karen Smith, David J. Weir, David J. Deehan

**Affiliations:** 1 Royal Infirmary of Edinburgh, Edinburgh, UK; 2 Present Address: Newcastle University, Newcastle upon Tyne, UK; 3 Newcastle upon Tyne Hospitals NHS Foundation Trust, Newcastle upon Tyne, UK

**Keywords:** Robotic, Knee, Arthroplasty, Outcome, Alignment, Balance, MAKO

## Abstract

**Background:**

Robotic-arm-assisted knee arthroplasty allows for more accurate component positioning and alignment and is associated with better patient-reported outcomes compared to manually performed jig-based knee arthroplasty. However, what is not known is whether the addition of an intra-articular sensor (Verasense^TM^) to aid intraoperative balancing of the total knee replacement (TKR) offers improved functional outcomes for the patient. The purpose of this research is to compare the outcomes of patients undergoing a conventional manual knee replacement to those undergoing TKR using robotic-assisted surgery and the Verasense^TM^ to optimise alignment and balance the knee joint, respectively, and assess the associated cost economics of such technology.

**Methods and analysis:**

This randomised controlled trial will include 90 patients with end-stage osteoarthritis of the knee undergoing primary TKR. Patients meeting the inclusion/exclusion criteria that consent to be in the study will be randomised at a ratio of 1:1 to either manual TKR (standard of care) or robotic-arm-assisted TKR with Verasense^TM^ to aid balancing of the knee. The primary objective will be functional improvement at 6 months following surgery between the two groups. The secondary objectives are to compare changes in knee-specific function, joint awareness, patient expectation and fulfilment, satisfaction, pain, stiffness and functional ability, health-related quality of life, cost-effectiveness, and gait patterns between the two groups. Ethical approval was obtained by the Tyne & Wear South Research Ethics Committee, UK. The study is sponsored by the Newcastle Hospitals NHS Foundation Trust.

**Discussion:**

This study will assess whether the improved accuracy of component positioning using the robotic-arm-assisted surgery and the Verasense^TM^ to aid balancing of the TKR offers improved outcome relative to standard manual jig-based systems that are currently the standard of care. This will be assessed primarily according to knee-specific function, but several other measures will also be assessed including whether these are cost-effective interventions.

**Trial registration:**

International Standard Randomised Controlled Trial Number ISRCTN47889316 10.1186/ISRCTN47889316. Registered on 25 November 2019

**Date and version for protocol:**

ROAM Protocol V1.0 (13-12-2018)

**Supplementary Information:**

The online version contains supplementary material available at 10.1186/s13063-021-05936-9.

## Background

Osteoarthritis (OA) of the knee is a degenerative disease and the prevalence increases with age [1]. Total knee replacement (TKR) is a cost-effective intervention for the management of end-stage OA of the knee with improvement in function and pain relief for the patient [2]. The rate of TKR continues to increase year on year in the UK which is thought to be due to growing elderly population that have increasing functional demands [3]. However, it is recognised that between 10 and 20% of patients after TKR are not satisfied with the outcome of their knee after surgery [4]. This results in a proportion of patients undergoing early (before 5 years) revision surgery for which most are due to component malalignment and instability [5]. Recent advances in technology has enabled robotic-assisted surgery to align the TKR within ± 1° with protection of the periarticular soft tissues and intra-operative pressure sensors (Verasense^TM^, OrthoSensor Inc. Dania Beach, FL, USA) can be used to objectively balance the TKR [6–8].

Robotic-assisted knee surgery has evolved over the last decade with increasing evidence of improved component alignment and patient outcomes when compared to manually performed knee surgery [9]. The most commonly used robotic-assisted TKR is the MAKO system. This requires a pre-operative CT scan of the patient’s hip, knee and ankle, which enables the computer navigation to accurately align the component using intra-operative trackers and mapping of the articular surface. The bone cuts, according to the pre-operative plan, are then made using the robotic-arm-assisted saw which also protects the soft tissue structures around the knee (haptic boundary). Non-randomised comparative cohort studies have shown a significant shorter hospital stay, improved early range of movement and function, and a greater rate of satisfaction with the robotic-arm-assisted TKR when compared to manual TKR [10, 11]. Despite the accuracy of the bone cuts to align the knee prosthesis correctly, the soft tissues, which can be scared and tight from the deformity, often need to be released to balance the knee as part of a measured resection technique [12, 13].

The majority of TKR are performed manually by the surgeon using jig-based systems and the knee is balanced by the ‘feel’ though a range of movement. It is recognised that manually performed TKR are misaligned in up to 30% of patients and during the saw cuts soft tissues around the knee may be damaged [14], which can disrupt the balance of the knee. In addition, the definition of a balanced TKR is variable and is dependent upon the surgeons ‘feel’ and hence is not a consistent measurable variable [15]. The Verasense^TM^ (OrthoSensor Inc. Dania Beach, FL, USA) enables the surgeon to objectively balance the TKR through soft tissue releases intra-operatively according to pressure readings within the knee, which has been shown to significantly improve the rate of patient satisfaction [8, 16].

The robotic-arm-assisted TKR has been demonstrated to be 100% accurate in achieving the planned alignment of the knee prosthesis [17], and the Verasense^TM^ (OrthoSensor Inc. Dania Beach, FL, USA) enables the surgeon to objectively balance the TKR with release of the peri-articular soft tissues or further bone cuts [8, 16]. It is hypothesised that robotic-assisted TKR will optimally align the prosthesis and the Verasense^TM^ (OrthoSensor Inc. Dania Beach, FL, USA) will balance the knee with releases of the peri-articular soft tissues which will result in an improved patient outcome when compared to conventional manual TKR.

Two potential reasons for patient dissatisfaction and poor functional outcome after TKR may be due to malalignment of the knee prosthesis and instability (not balanced) of the knee joint [4]. Robotic-assisted TKR offers accurate alignment of the prosthesis and the Verasense^TM^ (OrthoSensor Inc. Dania Beach, FL, USA) enables the surgeon to objectively balance the knee. Whether this improves the outcome of TKR is not known. Therefore, the purpose of this research is to compare the outcomes of patients undergoing a conventional manual knee replacement to those undergoing TKR using robotic-arm-assisted surgery and the Verasense^TM^ (OrthoSensor Inc. Dania Beach, FL, USA) to optimise alignment and balance the knee joint, respectively, and assess the associated cost economics of such technology in the National Health Service in the UK. The null hypothesis is that an optimally aligned and balanced TKR carried out with robotic-assisted surgery and the use of the Verasense^TM^ (OrthoSensor Inc. Dania Beach, FL, USA) does not improve early patient outcomes when compared to a TKR carried out manually using a jig-based system.

## Methods/design

### Objectives

The primary aim is to compare change in functional ability (measured by the functional component of the Western Ontario and McMaster Universities Osteoarthritis Index (WOMAC) [18]) from baseline to 6 months (early) following TKR between the two groups. The secondary outcomes are:

1. To compare changes in knee function in activities of daily living (measured by the Oxford knee score (OKS) [19]) from baseline to 3, 6 and 12 months
2. To compare changes in joint awareness (measured by the Forgotten Joint Score (FJS) [20]) from baseline to 3, 6 and 12 months
3. Pre-operative patient expectation and fulfilment at 6 and 12 months will be assessed using the Hospital for Special Surgery Knee Replacement Expectations Questionnaire (HSS) [21, 22]
4. To compare satisfaction using a validated outcome measure at 3, 6 and 12 months [23]
5. To compare pain, stiffness and functional ability (measured by the WOMAC [18]) at 3, 6 and 12 months
6. To compare the health related quality of life using the Euro Qol five dimensional (EQ5D-3L) [24] at baseline and 3, 6 and 12 months
7. To compare the cost effectiveness of the two interventions from a UK NHS perspective
8. To analyse any differences in gait patterns between the two groups

### Trial design

**Fig. 1:**
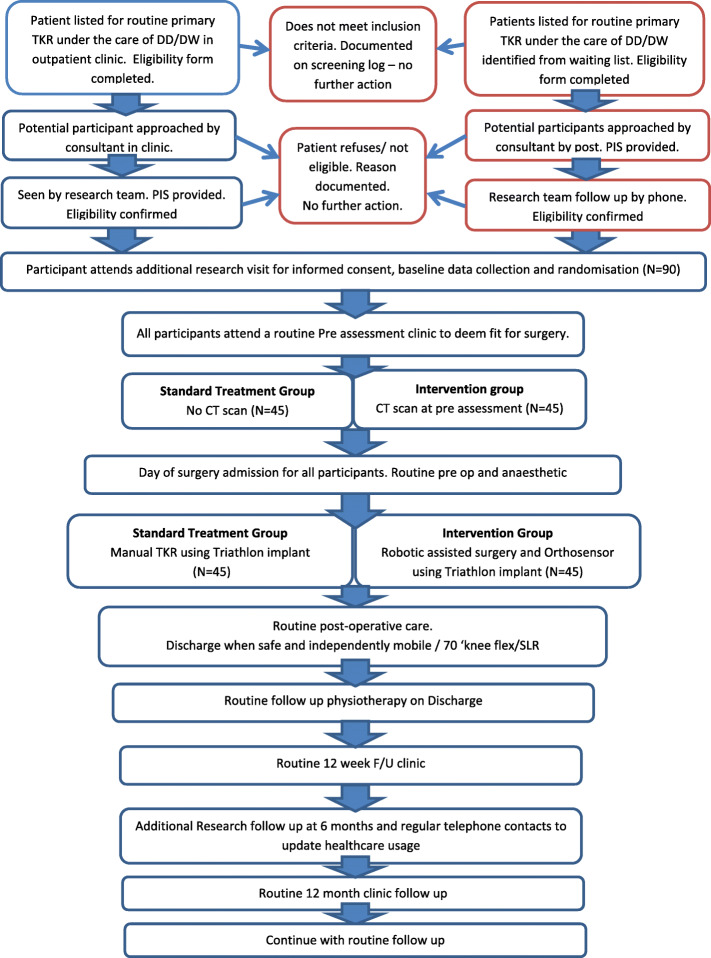
Flow diagram illustrating the patient screening, enrolment, randomisation and outcome assessment for the study cohort

**Fig. 2:**
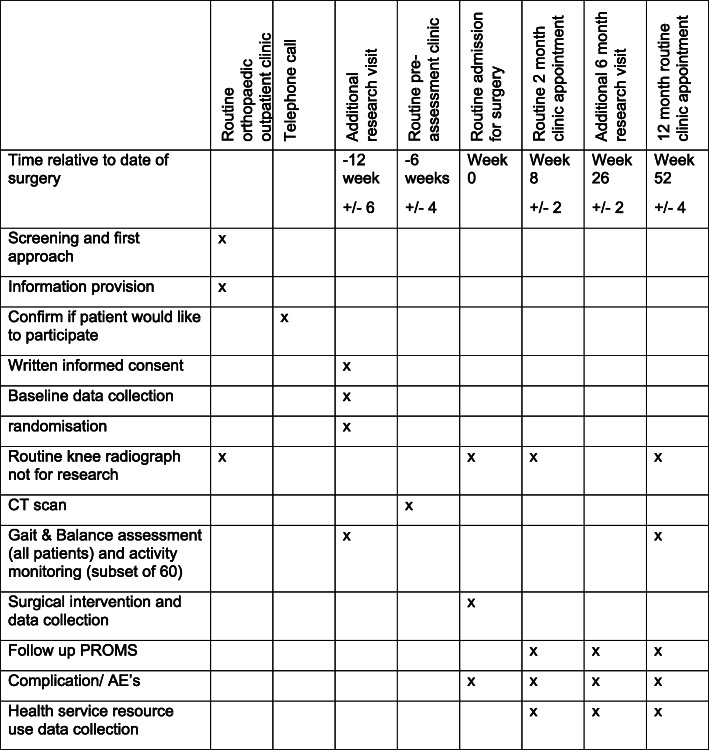
Schedule of enrolment and outcome assessments

A single site, prospective, parallel randomised controlled superiority trial will be conducted to compare the effectiveness of robotic-arm-assisted and sensor-assisted TKR to standard manual TKR. Participants will be randomly allocated 1:1 to either standard care or the intervention group. Participants randomised to standard care will undergo a conventional manual TKR. Participants randomised to the intervention group will undergo a TKR with the assistance of the MAKO robot and the use of sensors to optimise the balance and alignment of the TKR. Participants will be followed up at routine clinic appointments at 3 and 12 months post op with an additional postal questionnaire at 6 months post op (see flow chart of research activity below). After the 12-month follow-up, the participants will continue with the institution’s standard post-operative care. Screening, enrolment and assessments are illustrated in Figs. 1 and 2 and Additional file 1.

### Eligibility criteria

Participants listed for routine primary TKR for osteoarthritis under the care of the two participating surgeons at the institution will be screened for inclusion. The inclusion criteria are as follows: planned to undergo a primary TKR for end-stage osteoarthritis under the care of the two participating surgeons, age 45–85 at the time of listing for surgery, and suitable candidate for a cruciate retaining TKR (Triathlon prosthesis). The exclusion criteria are as follows: varus deformity of > 20° observed by consultant on examination, patient is unable to comply with the study protocol (incl. refusal for CT scan), female participants who is pregnant, lactating or planning pregnancy during the course of the study, requires patella resurfacing, inability to understand the patient information for the study, provide written informed consent or answer study questionnaires for cognitive or language reasons, or any other significant disease or disorder which, in the opinion of the Investigator, may either put the participants at risk because of participation in the study or influence the result of the study, or the participant’s ability to participate in the study.

### Recruitment

Patients listed for a routine primary TKR under the care of the participating surgeons will be screened by the treating clinician. A screening form will be completed to confirm name, age, hospital number, listed for routine primary TKR, suitable candidate for a cruciate retaining TKR (Triathlon prosthesis), no varus deformity of > 20°, capacity to provide informed consent and no pre-existing condition that limits function and potential outcome of surgery. After making the initial approach, the consultant will request the patient’s permission to be approached by the research team who will provide the patient information sheet, discuss the study in more detail and confirm eligibility. Patients will be encouraged to take the study information and discuss with their family and friends before making a decision to participate. The research team will reconfirm the eligibility criteria below and record that: the patient is able to comply with the study protocol (incl. refusal for CT scan) and that female participants are not pregnant, lactating or planning pregnancy during the course of the study.

Eligible patients are identified by the treating consultant surgeon and listed for surgery and a screening form is completed but are not approached at that time will be sent a letter of approach from the consultant along with the patient information sheet. The research team will then contact the patient to discuss the study in more detail and answer any questions. All eligible patients who agree to participate in the study will be invited to attend a research clinic to provide written informed consent and complete baseline assessments before randomisation.

Participants will be asked to attend a research appointment to provide written informed consent and complete a CRF and a number of pre-operative questionnaires recording their knee function, expectations and general health. Participants will be asked to carry out a gait assessment which involves walking over an instrumented walkway (Tekscan instrumented mat system, Tekscan Inc. Boston, USA) that is 3 m long plus 1 m either side, six times and standing still on an individual instrumented mat of the walkway for 1 min. After completing the baseline assessments participants will be randomised to one of two groups. Patients will be informed of their allocation and the research staff will reinforce the treatment allocation and arrange a CT for those randomised to the intervention group.

All participants will attend a routine pre-assessment clinic 4–6 weeks prior to surgery to assess fitness for surgery. Pre-assessment staff will be notified of the patient’s inclusion in the study and their group allocation. Participants randomised to the robotic-assisted surgery group will be required to have an additional CT scan which may be arranged to coincide with this appointment. At this routine clinical appointment, the research team will ensure all baseline data is complete and ensure the patient has no concerns. The CT scan will be anonymised using a dedicated study code and transferred to Stryker using an encrypted data drop box. The CT scan will be reconfigured and transferred back using an encrypted data drop box. This data will be used by the MAKO technician to plan the individuals’ surgery. This process is standard practice in hospitals across the UK already using the MAKO for robotic-assisted knee surgery.

All participants will be admitted in line with routine practice. Surgical data and hospital discharge data will be collected from source data to complete the CRF during the inpatient stay. No additional research assessments will be carried out at this point.

All participants will attend a routine 3-month post-surgery assessment with the clinical team including knee radiographs. Participants will also be seen by the research team to complete the relevant patient-reported questionnaires, Health service resource use and complications data forms.

All participants will be asked to attend an additional 6-month post-surgery research visit to complete the relevant patient-reported questionnaires, health service resource use and complications data forms. The gait assessment carried out at baseline will be repeated.

A member of the research team will contact the participant by telephone at 9 months following surgery to complete the health service resource use questionnaire as this only captures data over the previous 3 months.

All participants will attend a routine 12-month post-surgery assessment with the clinical team including knee radiographs. Participants will also be seen by the research team to complete the relevant patient-reported questionnaires, health service resource use and complications data forms. A gait analysis assessment will also be carried out.

### Consent

Written and verbal versions of the participant information and informed consent will be presented to the participants by the research team. This will explain no less than the exact nature of the study, the implications and constraints of the protocol and any risks involved in taking part. It will be clearly stated that the participant is free to withdraw from the study at any time for any reason without prejudice to future care, and with no obligation to give the reason for withdrawal. The participant will be encouraged to take the study information home and have the opportunity to question the Investigator, their GP or other independent parties to decide whether they will participate in the study.

Participants who decide to take part will be invited to an additional research visit to provide written informed consent. Written informed consent will be obtained by means of participant dated signature and dated signature of the person who presented and obtained the informed consent. The person who obtained the consent will be suitably qualified and experienced member of the research team and have been authorised to do so by the Chief/Principal Investigator. Copies of the signed informed consent will be given to the participants as well as filed in the medical notes. The original signed form will be retained at the study site in the TMF.

The participant must personally sign, print their name and date the latest approved version of the informed consent form before any study-specific procedures are performed. If for whatever reason the patient is unable to print their name or date the consent form, this can be completed by the researcher taking consent, at the patient’s request. It must be fully documented on the consent form that the researcher has taken this action, with the reason why also documented. However, the researcher must *not* sign the consent form on behalf of the patient; the patient must always sign the consent form.

### Allocation

Patients are allocated once they have been screened and have given informed consent to participate in the trial. Subject numbers will be assigned sequentially as each subject enters the study. Participants will be randomised on a 1:1 ratio using the Sealed Envelope software (www.sealedenvelope.com) after consent and baseline data collection is complete to reduce the risks of selection bias. Randomisation will be carried out in random sized equally split blocks to allow even balancing of the groups and avoid selection potential bias. Patients will be allocated to standard treatment (manual TKR) or intervention group (robotic-arm- and sensor-assisted TKR). It will not be possible to conceal the allocation of treatment from the patient, or the clinicians. Research staff completing follow-up assessments and data analysts will be blinded to the participant’s allocation to reduce potential bias.

### Outcome measures

The primary goal of joint replacement surgery is to restore function and reduce pain, the outcome measures have been chosen to reflect these factors. The primary outcome will be changed in functional ability measured by the WOMAC [18] questionnaire from baseline to 6 months following TKR. The WOMAC^18^ questionnaire consists of 24 self-administrated questions with pain, stiffness and function subscales. The score is validated for patients with knee OA to detect changes in functional ability.

The following outcome measures will be used to assess changes in activity levels, joint awareness, and satisfaction and health status as secondary outcomes. Change in activity participation from baseline to 3, 6 and 12 months following knee replacement will be assessed using the OKS [19] which consists of twelve questions assessed on a Likert scale with values from 0 to 4; a summative score is then calculated. In addition, a higher functioning supplementary questionnaire consisting of a further eight questions will be used. This was designed to assess activity participation for patients that may achieve the ceiling score in the OKS [25]. Change in joint awareness from baseline to 3, 6 and 12 months following knee replacement surgery will be assessed using the FJS which is designed to assess joint awareness in hips and knees during various activities of daily living [20]. It uses a 5-point Likert response format, consisting of 12 equally weighted questions with the raw score transformed to range from zero to 100 points. In previous studies, the score has shown good reliability and convergent validity [20], performed well in known-group comparisons, and was found to be sensitive to change over time [26]. Patient expectation pre-operative and fulfilment will be assessed at 3, 6 and 12 months following knee replacement surgery. The HSS is a validated measure of patient pre-operative expectations of surgery [21, 22, 27]. The level of patient expectation is indicated on a 5-point Likert scale as ‘very important’, ‘somewhat important’, ‘a little important’, ‘I do not expect this’ or ‘this does not apply to me’ [21]. After surgery, patients will complete a similar expectation questionnaire, but are asked whether the same expectations had been fulfilled, which again is assessed on a 5-point Likert scale as ‘greatly’, ‘a lot’, ‘a little’, ‘I did not expect this’ or ‘this did not apply to me’ [22]. Patient satisfaction at 3, 6 and 12 months following hip replacement surgery and will be assessed following surgery by asking four questions with a different focus:

- ‘Overall how satisfied are you with the results of your hip replacement surgery?’
- ‘How satisfied are you with the results of your hip replacement surgery for improving your ability to do housework or yard work (such as cooking, cleaning, or gardening and raking leaves)?’
- ‘How satisfied are you with the results of your hip replacement surgery for improving your ability to do recreational activities (such as taking walks, swimming, bicycling, playing golf, dancing, going out with friends)?’
- ‘How satisfied are you with the results of your hip replacement surgery for relieving your pain?’

The response to each question will be recorded using a 4-point Likert scale: very satisfied, somewhat satisfied, somewhat dissatisfied and very dissatisfied. These questions and the 4-point Likert assessment have been validated and demonstrated to be reliable to measure satisfaction following primary hip replacement surgery [23]. Change in pain, stiffness, and functional ability from baseline to 3, 6 and 12 months following surgery will be assessed using the subscales of the WOMAC [18] questionnaire. Change in patient-reported quality of life from baseline to 3, 6 and 12 months following knee replacement surgery will be assessed using the EQ-5D-3L general health questionnaire [28] which evaluates five domains (-5D), which include mobility, self-care, usual activities, pain/discomfort and anxiety/depression. This is a two page questionnaire that consists of five dimensions, with the responses recorded at three levels (3L) of severity (no problems, some problems or extreme problems). The second page consists of a standard vertical 20cm visual analogue scale (EQ-5D VAS) which is transformed to a scale of 0 to 100 measuring current health-related quality of life. Each patient’s health state, derived from the EQ-5D, will be measured before and after their surgery to determine the change in their health gain or loss after their knee replacement surgery. The health state will then be multiplied by the time spent in that state to derive the QALYs gained or lost. The cost per QALY will then be calculated by dividing the cost of the procedure by the QALYs gained after TKR.

A cost-utility analysis will be undertaken from a UK NHS perspective up to 12 months following knee replacement and also projected over 5 and 10 years and a lifetime horizon. A health service resource use questionnaire will be completed by the patient at the 3-, 6- and 12-month research assessment. The questionnaire collects data on primary, secondary and community care and associated with the knee replacement over the previous 3 months. An additional telephone follow-up will be carried out at 9 months where the research team will complete the questionnaire with the participant to ensure the data is complete for the 12-month follow-up period. Inpatient and surgical data will be collected on the case report forms (CRFs) and complications will be recorded at each visit.

Gait patterns will be analysed at baseline and 3 months following surgery. Participants will complete a gait assessment to record spatiotemporal characteristics of their walking pattern prior to surgery. The gait assessment will consist of the patient walking over a 3-m instrumented walkway (Tekscan Inc., Boston, USA), including a metre at either end of the walkway to allow for acceleration and deceleration, a total of 5 m.

### Defined end of trial

The end of trial is the date of the 12-month follow-up visit of the last participant. Each participant has the right to withdraw from the study at any time. In addition, the investigator may discontinue a participant from the study at any time if the investigator considers it necessary for any reason including ineligibility (either arising during the study or retrospective having been overlooked at screening); significant protocol deviation, i.e. intervention not used as intended at time of surgery; significant non-compliance with treatment regimen or study requirements; an adverse event which requires discontinuation of the study medication or results in inability to continue to comply with study procedures; consent withdrawn; or lost to follow-up.

Participants who wish to withdraw consent for the trial or whose participation from the trial is discontinued will have anonymised data collected up to the point of that withdrawal of consent included in the analyses unless the participant specifically asks for all data collected to be destroyed. No additional data will be collected from the participant. The reason for withdrawal will be recorded in the CRF. Participants who withdraw once they have been randomised will not be replaced. If the participant is withdrawn due to an adverse event, the investigator will arrange for follow-up visits or telephone calls until the adverse event has resolved or stabilised.

At the end of the trial, patient will resume standard practice of care of the study centre in place at that time for follow-up of routine TKR.

### Surgical intervention

All participants will receive a Triathlon TKR with a highly crossed linked (X3) cruciate retaining polyethylene insert. This is the current standard care for both participating surgeons. All participants will receive standard post op nursing and rehabilitation care. Prior to discharge from hospital patients will undergo the routine physiotherapy and occupational assessments for activities of daily living and equipment will be provided as routine.

Patients randomised to standard will receive a conventional manual Triathlon TKR with a highly crossed linked (X3) cruciate retaining polyethylene insert. In a manually performed TKR, the surgeon will make an incision down the front of the knee and move the knee cap to one side to allow access to the knee joint. The surgeon will then make bone cuts using a manual jig-based system and a hand held saw to prepare the bone surfaces for the implant. A measured resection technique will be employed with 8-mm resection from the distal femur and 9mm from the proximal tibia with a 3° slope. Both surgeons will use a conventional jig alignment technique for intramedullary referencing for the femur and extra medullary referencing for the tibia. Sizing of the femoral component and rotation (using Whiteside’s line [29]) will be performed manually after intra-operative assessment. Once the implant is in position the knee is then balanced by the ‘feel’ though a range of movement and soft tissue releases will be performed as required to balance the knee in flexion and extension.

The intervention group (undergoing robotic-arm-assisted surgery) will require a pre-operative CT scan as part of the planning process for robotic-assisted surgery. The information from the CT scan will be used to create a 3D model of the patient’s bony anatomy to plan the positioning and sizing of the implant. The research team will make every effort to coordinate the CT scan with the patient’s routine pre-assessment clinic to limit the requirement for additional hospital visits. The CT scan will be anonymised using a dedicated study code and transferred to Stryker using an encrypted data drop box. The CT scan will be reconfigured and transferred back using an encrypted data drop box. This data will be used by the MAKO technician to plan the individuals’ surgery. The participants will receive the same Triathlon TKR with a highly crossed linked (X3) cruciate retaining polyethylene insert and the same surgical approach to the knee joint will be employed. Instead of using a manual jig-based system and a handheld saw to prepare the bone surfaces for the implant, the MAKO robotic-arm will be used by the surgeon to cut the bone at the required alignment. The MAKO is a robotic arm that the surgeon guides to cut the diseased bone instead of using a manual jig and the handheld saw. The information from the CT scan will be used to create a 3D model of the patient’s bony anatomy and will used to plan the positioning of the implant. During the operation, trackers (markers for the robot to assess where the knee is in space) will be positioned on the tibia and femur using two treaded pins (4mm) through a small incision (2cm) a hands width above and below the joint. Once the trackers are in place, registration of the knee joint surface is performed. The specified bone cuts are then performed using the robotic arm. The specified bone cuts will be 5° of valgus and neutral flexion/extension for the femoral component and zero varus/valgus with 3° of posterior slope for the tibial component relative to the mechanical axis. When the implant is in place, the Verasense^TM^ (OrthoSensor Inc. Dania Beach, FL, USA) will then be inserted into the knee joint. Pressures in the medial and lateral compartments will be recorded throughout a range of movement (extension to flexion). Soft tissue releases will be performed as required to balance the knee, defined as a pressure difference of less than 15Ibs in medial and lateral compartments, throughout a range of motion [16].

All patients will receive the same standard of inpatient care and rehabilitation and discharge advice to progress mobility and standard exercises. Data will be collected from source data regarding LOS and discharge details. Data regarding health care usage and equipment will be collected using the modified CSRI completed at follow-up clinics and interim phone call follow-ups. The research team will contact the patient by phone and at scheduled research visits to optimise completion over the study period.

### CT scan

CT is a procedure that uses x-ray equipment to create cross-sectional pictures of bony anatomy. During the scan, the participant will lie on a narrow platform bed as it slides through the scanner, which is shaped like a large donut. A CT scanner creates clear and detailed pictures of bones. CT scans involve radiation and there are small risks associated with radiation exposure. These are described in patient information leaflet. The CT scan is an essential component of the research study and provides the anatomical information required for the MAKO robot to accurately perform the bone cuts during surgery. If a patient is unable to undergo a CT scan for any reason, then they are unable to participate in the research study.

### Safety reporting

The risks for the two groups are those particular to TKR and from an operative intervention. This includes infection, thrombosis, stiffness, instability, pain, scar sensitivity, loss of function, nerve or vessel injury, requirement for revision with attendant time off work. General risks include cardiorespiratory risk, angina, MI, stroke, pneumonia, risks from prolonged recumbency, urinary infection, skin ulcers. Potential risks and steps taken to minimise these risks are outline in the PIL. In addition it is anticipated that those patients with patients who are randomised to use of the robotic instrumentation may require a longer procedure of up to 5 min [10], and there is a theoretical increased risk of infection. There is also a small (1%) risk of sustaining a fracture through a tracker pin (a threaded pin inserted into the bone so the robot can establish the position of the bone in time and place) [30, 31]. Tracker pins are routinely used for navigated TKR surgery and this increased risk is accepted to enable more accurate placement on the knee prosthesis. However, to our knowledge, there is no reported case of a fracture through a tracker pin site when used for robotic TKR surgery. There is also a small risk (1%) of wound infection at the site of tracker pins, but again these are established and accepted in navigation TKR surgery [32].

An adverse event (AE) is any untoward medical occurrence in a patient or other clinical investigation participant taking part in a trial of a medical device, which does not necessarily have to have a causal relationship with the device under investigation. An AE can therefore be any unfavourable and unintended sign (including an abnormal laboratory finding), symptom or disease temporally associated with the use of the device, whether or not considered related to the device.

An adverse device effect (ADE) is all untoward and unintended responses to the medical device. The phrase ‘responses to a medical device’ means that a causal relationship between the device under investigation and an AE is at least a reasonable possibility, i.e. the relationship cannot be ruled out. All cases judged by either the reporting medically qualified professional or the sponsor as having a reasonable suspected causal relationship to the device qualifies as a device effect. This also includes any event resulting from insufficiencies or inadequacies in the instruction for use or deployment of the device and includes any event that is a result of a user error.

A device deficiency (DD) is inadequacy of a medical device related to its identity, quality, durability, reliability, safety, or performance, such as malfunction, misuse or use error and inadequate labelling.

‘Serious’ in serious adverse event (SAE) means an adverse event resulting the following: death, in-patient hospitalisation or prolonged hospitalisation (defined as an inpatient admission, regardless of length of stay). However, hospitalisation for pre-existing conditions (including elective procedures that have not worsened) or additional elective hospitalisation does not constitute a SAE. A life-threatening illness or injury in the definition of ‘serious’ refers to an event in which the participant was at risk of death at the time of the event; it does not refer to an event which hypothetically might have caused death if it were more severe. Persistent or significant disability or incapacity. Congenital anomaly or birth defect. Other serious events that may jeopardise the patient and may require medical or surgical intervention to prevent one of the other five listed outcomes. This includes device deficiencies that might have led to an SAE if suitable action had not been taken, intervention had not been made or if circumstances had been less fortunate.

To ensure no confusion or misunderstanding of the difference between the terms ‘serious’ and ‘severe’, which are not synonymous, the following note of clarification is provided: The term ‘severe’ is often used to describe the intensity (severity) of a specific event (as in mild, moderate, or severe myocardial infarction); the event itself, however, may be of relatively minor medical significance (such as severe headache). This is not the same as ‘serious’, which is based on patient/event outcome or action criteria usually associated with events that pose a threat to a participant’s life or functioning. Seriousness (not severity) serves as a guide for defining regulatory reporting obligations.

A serious adverse device effect (SADE) is any untoward medical occurrence seen in a patient that can be directly related to the Stryker product resulting in resulting in any of the characteristics or led to characteristics of a serious adverse event. SADE is also any event that may have led to these consequences if suitable action had not been taken or intervention had not been made or if a circumstance has been less opportune. All cases judged by either the reporting medically qualified professional or the sponsor.

Anticipated serious adverse device effect (ASADE) which by its nature, incidence, severity or outcome has been previously identified in the current version of the risk analysis report or Investigator Brochure.

Unanticipated adverse device effect (UADE) is any serious adverse device effect on health or safety or any life-threatening problem or death caused by or associated with a device, if that effect, problem or death was not previously identified in nature, severity or degree of incidence in the investigational plan or application (including a supplementary plan or application), or any other unanticipated serious problem associated with a device that related to the rights, safety or welfare of the subject.

Tables 1 and 2 categorise the adverse event according to whether it is related to the device. Each AE will be assessed for seriousness, severity, causality and expectedness by an appropriately trained and delegated investigator. The Investigator, or an appropriately trained designee, should make an assessment of seriousness using the criteria in Table 3.

**Table 1:** Classification of adverse event according to relation to the device (robot)

Adverse Events	Non-device related	Device or procedure related	
**Non-serious**	AE	ADE	
**Serious**	SAE	SADE	
		Anticipated	Unanticipated
		ASADE	USADE

**Table 2:** Device deficiency category according to SADE

Device deficiency	Could lead to a SADE	Would not lead to a SADE
**Category:**	Device deficiency with SADE potential	Device deficiency without SADE potential

**Table 3:** Classification criteria for the intensity of the adverse event

Intensity	Description
Mild	An event easily tolerated by the patient, causing minimal discomfort and not interfering with everyday activities
Moderate	An event sufficiently discomforting to interfere with normal everyday activities
Severe	An event that prevents normal everyday activities

The expectedness of the AE will be assessed by the CI or an appropriately trained designee using the list of expected events below. This list is based on current clinical knowledge of expected intra- and post-operative complications of TKR. Intra-operative complications: not able to proceed with expected implant (in this case triathlon TKR), soft tissue damage, neurovascular damage, fracture, patients becomes medically unwell and cannot perform TKR, blood loss and general medical complications, e.g. heart attack, stroke. Post-operative complications: infection (superficial and deep implant infection), deep vein thrombosis and pulmonary embolus, stiffness, swelling, numbness, neurovascular complications, wound breakdown, fracture and general health problems, e.g. stroke, heart attack, kidney failure and potential loss of limb and life.

The relationship between the investigational medical device (including comparator treatments) and the occurrence of each AE must be assessed and categorised by the Chief Investigator (or delegate). Related events are those that are related to the administration of the medical device or study procedures. Each AE will be categorised as (1) not related (no relationship with the investigational device; other factor(s) certainly or probably causative) or (2) possibly related (the nature of the event, underlying medical condition, concomitant medication or temporal relationship make it possible that the AE has a causal relationship to the device).

All AE’s occurring during the study observed by the investigator or reported by the participant, whether or not attributed to the device under investigation will be recorded on the CRF as specified in the protocol. All ADE’s will be recorded in the CRF. The following information will be recorded: description, date of onset and end date, severity, assessment of relatedness to device, other suspect drug or device and action taken. Follow-up information should be provided as necessary. The relationship of AEs to the device will be assessed by a medically qualified investigator or the sponsor/manufacturer and will be followed up until resolution or the event is considered stable.

All ADE that result in a participant’s withdrawal from the study or are present at the end of the study should be followed up until a satisfactory resolution occurs. All DDs should be reported to NUTH via the safety reporting inbox: tnu-tr.safetyreporting@nhs.net

All SAEs/SADEs/ASADEs/UADEs will be reported to the CI in the first instance, who will determine seriousness, relatedness and expectedness. The event will then be reported to the sponsor/legal representative NUTH R&D Regulatory Compliance Team at tnu-tr.safetyreporting@nhs.net within one working day of the investigator team becoming aware of them. For a related serious adverse event (SAE) the investigator will complete a Stryker ‘Product Experience Report’ form *(**www.stryker.com**product experience report)* or inform a Stryker employee*.*

Reports of related and unexpected SAEs will be submitted to ethics within 15 days of the Chief Investigator becoming aware of the event, using the NHS HRA Non-CTIMP Safety Report to REC form (Report of Serious Adverse (non-CTIMP) Form). Related and unexpected events must also be reported through the NUTH DATIX Incident Reporting System by a member of the research team. USADEs occurring in NUTH sponsored studies must also be reported via the electronic incident reporting system, DATIX. The incident type should be entered as ‘Research Incident/Accident’, Directorate as ‘Medical Director’s Directorate’, Speciality as ‘Research Development and Governance’, Site as ‘Regent Point’ and Location as ‘Research Buildings’. The event must also be assessed by the CI to determine whether or not it constitutes a serious incident (SI). This is defined as an event or incident that resulted in unexpected or avoidable death, or serious harm, to one or more patients, staff, visitors or members of the public. If the event has been classed as (or is suspected to be) a SI, an NUTH NHS Foundation Trust manager will inform the Director of Quality and Effectiveness, via. the Clinical Governance and Risk Department (CGARD), as soon as possible by telephone. This is detailed in the NUTH FT ‘Serious Incident Reporting and Management Policy’.

If an ADE is defined as serious (i.e. a SADE) or a DD that could have led to a SADE/USADE, the investigator will quarantine the device as soon as possible. This involves segregating the device from other equipment and labelling it as ‘not for use’ with relevant contact details of the research team. The device and all associated items (including relevant packaging materials) should be quarantined. They should not be repaired, discarded, or returned to the manufacturer without agreement from Sponsor.

### Statistical analysis

Statistical analysis will be performed using Statistical Package for Social Sciences version 17.0 (SPSS Inc., Chicago, IL, USA). Parametric and non-parametric tests will be used as appropriate to assess continuous variables for significant differences between groups. Changes in key outcomes (WOMAC, OKS, EQ-5D) from baseline to 3, 6 and 12 months following surgery will be analysed using analysis of variance (ANOVA) and adjusted for multiple testing to compare the two intervention arms. The data is expected to demonstrate a normal distribution and this will be assessed prior to analysis. A *p*-value of <0.05 will be defined as significant for the primary outcome and a *p*-value of <0.01 will be defined as significant for the secondary outcomes to adjust for multiple testing.

An economic evaluation will be conducted from an NHS perspective. Data collection from the study will also focus on estimating the cost of the interventions and subsequent use of services. Analyses will be carried out from an NHS perspective and will include the capture of resource use from secondary and primary/community care. All relevant costs associated with providing the interventions will be included including length of stay. All unit costs will be derived using routine data sources and study specific estimates. Costs in the follow-up period will include use of secondary care services, e.g. inpatient stays and outpatient visits, and primary/community care services, e.g. general practice visits, district nurse visits and prescription costs incurred over the follow-up period. This data will be collected using a health service utilisation patient diary. Data on use of services will be combined with appropriate unit costs to produce a cost for each trial participant. From these, a mean cost per intervention will be calculated. Health-related quality of life will be measured using EQ-5D-3L which is a very simple measure which patients complete at the start and end of treatment. It comprises five dimensions of health: mobility, ability to self-care, ability to undertake usual activities, pain and discomfort, and anxiety and depression. The cost-effectiveness analysis of the two types of surgery will be based on incremental cost of a quality-adjusted life year (QALY). QALYs will be estimated using the area under the curve approach. The results will be presented as point estimates of mean incremental costs and QALYs. The results of the analyses will include deterministic and stochastic sensitivity analysis, presented as point estimates and cost-effectiveness acceptability curves (CEACs).

### Sample size

A power calculation was performed using the functional component of the WOMAC [18] as the primary outcome. Using the defined minimal clinically important difference in the WOMAC of 15 points [33], a standard deviation (SD) of 26.5, and an alpha 0.05 with a power of 80% to demonstrate a superior outcome in the intervention group (one tailed analysis), it was determined that 80 patients would need to be recruited (40 in each arm). Loss to follow-up data from other surgical arthroplasty studies in the institution with a similar follow-up regime were used to estimate a loss to follow-up of 10%. On this basis, 90 patients will be randomised to ensure the study achieves power.

### Data recording and record keeping

Data will be collected by the site researcher (baseline collection), clinicians delivering the interventions and participants (health utility and assessment questionnaires). All data requested on the CRF must be recorded. All missing data must be explained. If a space on the CRF is left blank because the procedure was not done or the question was not asked ‘N/D’ will be written. If the item is not applicable to the individual case, ‘N/A’ will be written. If the data item is unknown, ‘NK’ will be written. If a data item has not been recorded on source data then ‘NR’ will be written. All entries should be printed legibly in black ink. If any entry error has been made, to correct such an error, a single straight line should be drawn through the incorrect entry and the correct data entered above it. All such changes must be initialled and dated and errors should not be erased or whited out. For clarification of illegible or uncertain entries, the clarification should be printed above the item, then initialled and dated.

All trial data will be entered on to paper CRFs and subsequently inputted into the trial database by the research team. In accordance with the ICH GCP (Section 5.5) [61], electronic data entry systems will be validated and standard operating procedures for data entry will be maintained.

The study will be conducted in accordance with the current approved protocol, ICH GCP, relevant regulations and standard operating procedures. Regular monitoring will be performed according to ICH GCP. Data will be evaluated for compliance with the protocol and accuracy in relation to source documents. Following written standard operating procedures, the monitors will verify that the clinical trial is conducted, and data are generated, documented and reported in compliance with the protocol, GCP and the applicable regulatory requirements. Monitoring and auditing will be undertaken in accordance with the sponsors policies and procedures and complying with Department of health Research Governance Framework for Health and social care (2005).

The chief Investigator and other key staff will attend site initiation training, coordinated by the research team, which will incorporate elements of trial-specific training necessary to fulfil the requirements of the protocol. Training will consist of reviewing the trial protocol, recruitment, consent, randomisation, follow-up, trial procedures, intervention training as applicable, trial arrangements, data protection and data handling.

## Dissemination policy

The results of the study will be presented at local, national and international meetings. Publication of the study will likely take part in several stages with the main focus being the primary outcome. However, we suspect there will be too much data to present in one encompassing manuscript and several publications relating to the secondary outcomes will be published.

## Discussion

This study aims to assess whether the improved accuracy of implant alignment using robotic arm-assisted surgery and the Verasense^TM^ (OrthoSensor Inc. Dania Beach, FL, USA) aids the surgeon to balance the TKR intraoperatively results in a greater improvement in the early knee-specific outcome. In addition, the secondary outcomes assessed in this study will enable quantification of the patient’s expectations fulfilment, improvement in their general health, restoration normal gait and patient satisfaction. Furthermore, the study aims to assess whether robotic arm-assisted TKR is a cost-economically viable treatment within the National Health Service in the UK.

The major limitation of the study design is the non-blinding of the patients to the intervention that they will receive, and this may bias the patient-reported outcomes measures assessed. The reason for this is due to the need for a pre-operative CT scan in those patients randomised to undergo robotic arm-assisted TKR and associated radiation exposure; therefore, patients needed to be informed of this prior to enrolling to the study. The authors considered performing placebo CT scans for those undergoing manual TKR using jig-based systems to enable blinding of the patients, but this was not possible due to the fact patients would be exposed to a significant dose of radiation that would be of no benefit to the patient. The assessors were blinded to the intervention arm, but the majority of the outcome measures assessed in this study are subjective patient reported outcomes.

The final limitation was the use of the minimal clinically important difference of 15 points to power the study to the functional component of the WOMAC score. This was taken from Escoban et al. [33] whom defined the MCID to range from 15 to 22 points following TKR, and therefore, 15 points was taken as the lower limit and used to power the study. However, recently Clement et al. [34] have demonstrated the MCID may be as low as 9 points for the functional component of the WOMAC 1 year following TKR. Substituting this MCID of 15 for 9 and using a SD of 18 points (as observed by Clement et al. [34] for change in functional score) at least 102 patients would need to be recruited (51 in each treatment arm). Funding and study designed and infrastructure were all in place prior to the publication of this paper, and therefore, the study is continuing to aim to show a statistical 15 point or more difference, but it should be acknowledged a difference less than this may be non-significant but may be clinically significant if the difference is 9 points or more.

## Trial status

Protocol version 1.0 date 13/12/2018. Patient recruitment date: May 2019. Estimated completion of recruitment date: May 2021. Estimated completion of final follow-up date: December 2022.

## Supplementary Information

**Additional file 1.** SPRINT template for the schedule of enrolment, interventions, and assessments.

## Data Availability

The datasets used and/or analysed during the current study are available from the corresponding author on reasonable request.

## Abbreviations

AE: Adverse event
ADE: Adverse device effect
CI: Chief Investigator
CRF: Case report form
CRO: Contract Research Organisation
CT: Clinical trials
CT scan: Computed tomography scan
CTA: Clinical Trials Authorisation
EC: Ethics Committee (see REC)
FJS: Forgotten Joint Score
GCP: Good Clinical Practice
GP: General Practitioner
ICF: Informed Consent Form
ICH: International Conference of Harmonisation
IEC: Independent Ethics Committee
IRB: Independent Review Board
MHRA: Medicines and Healthcare products Regulatory Agency
NHS: National Health Service
NRES: National Research Ethics Service (previously known COREC)
OA: Osteoarthritis
OKS: Oxford knee score
PI: Principal Investigator
PIL: Participant/ Patient Information Leaflet
R&D: NHS Trust R&D Department
REC: Research Ethics Committee
SAE: Serious adverse event
SADE: Serious adverse device effect
SIL: Subject Information Leaflet (see PIL)
SOP: Standard operating procedure
TKR: Total knee replacement
TMF: Trial Master File
UADE: Unanticipated adverse device effect
WOMAC: Western Ontario and McMaster Universities Osteoarthritis
