# Supplementary material for: Robotic- and orthosensor-assisted versus manual (ROAM) total knee replacement: a study protocol for a randomised controlled trial
Source: Trials. 2022 Jan 22;23:70. doi: 10.1186/s13063-021-05936-9 (PMC8783439; doi:10.1186/s13063-021-05936-9)
Supplement: Supplementary file 1 — Additional file 1. SPRINT template for the schedule of enrolment, interventions, and assessments. [file 13063_2021_5936_MOESM1_ESM.doc]

Appendix 1. SPRINT template for the schedule of enrolment, interventions, and assessments.

|  | **STUDY PERIOD** | | | | | | | |
| --- | --- | --- | --- | --- | --- | --- | --- | --- |
|  | **Enrolment** | **Allocation** | **Post-allocation** | | | | | **Close-out** |
| **TIMEPOINT (weeks)** | ***Out patient clinic**** | **-12 (+/-6)** | ***-6*** | ***0*** | ***12*** | ***26*** | ***38*** | ***52*** |
| **ENROLMENT:** |  |  |  |  |  |  |  |  |
| **Eligibility screen & information** | X |  |  |  |  |  |  |  |
| ***Confirm willing to participate*** |  | X |  |  |  |  |  |  |
| **Informed consent** |  | X |  |  |  |  |  |  |
| **Allocation** |  | X |  |  |  |  |  |  |
| **INTERVENTIONS:** |  |  |  |  |  |  |  |  |
| ***Manual TKA*** |  |  |  | X |  |  |  |  |
| ***Robotic TKA]*** |  |  |  | X |  |  |  |  |
| **ASSESSMENTS:** |  |  |  |  |  |  |  |  |
| ***Baseline data*** |  | X |  |  |  |  |  |  |
| ***Knee Radiograph*** | X |  |  | X | X |  |  | X |
| ***CT Scan*** |  |  | X |  |  |  |  |  |
| ***Gait assessment*** |  | X |  |  |  | X |  |  |
| ***PROMS*** |  | X |  |  | X | X | X | X |
| ***Joint compartment pressures*** |  |  |  | X |  |  |  |  |
| ***Complications and adverse events*** |  |  |  | X | X | X | X | X |
| ***Health service use data collection*** |  |  |  | X | X | X | X | X |

*Routine orthopaedic outpatient clinic where eligible patient will be identified. The time at which this occurs prior to surgery (time 0) will be variable depending on waiting list pressures and the patients availability.

PROMS: patient reported outcome measures
